# Crystal structure of hexa­kis­(dimethyl sulfoxide-κ*O*)cobalt(II) bis­[tri­chlorido­(quinoline-κ*N*)cobaltate(II)]

**DOI:** 10.1107/S2056989018001652

**Published:** 2018-02-07

**Authors:** Tyler K. Brescia, Kaltrina Mulosmani, Shivani Gulati, Demosthenes Athanasopoulos, Rita K. Upmacis

**Affiliations:** a Department of Chemistry and Physical Sciences, Pace, University, New York, NY 10038, USA; b Department of Chemistry, Columbia University, New York, NY 10027, USA

**Keywords:** crystal, cobalt, quinoline, dimethyl sulfoxide

## Abstract

There are few reports that describe crystal structures of compounds containing cobalt complexed to either dimethyl sulfoxide (Me_2_SO) or quinoline (C_9_H_7_N). The title compound, [Co(C_2_H_6_OS)_6_][CoCl_3_(C_9_H_7_N)]_2_, is a cobalt salt in which the metal ion is complexed to both Me_2_SO and quinoline. In particular, we observed that anhydrous cobalt(II) chloride reacts with quinoline in Me_2_SO to form a salt that is to be formulated as [Co^II^(Me_2_SO)_6_]^2+^{[Co^II^Cl_3_quinoline]_2_^−^}. The Co^II^ atom in the cation portion of this mol­ecule lies on a inversion center and is bound to the O atoms of six Me_2_SO moieties in an octa­hedral configuration, while the Co^II^ atom in the anion is attached to three chloride ligands and one quinoline moiety in a tetra­hedral arrangement.

## Abstract 2

Anhydrous cobalt(II) chloride reacts with quinoline (C_9_H_7_N) in dimethyl sulfoxide (Me_2_SO) to form the novel complex salt [Co^II^(Me_2_SO)_6_][Co^II^Cl_3_quinoline]_2_. The compound comprises an octa­hedral homoleptic Me_2_SO-solvated cobalt(II) cation and a tetra­hedral cobaltate(II) anion attached to three chloro ligands and one quinoline moiety.

## Chemical context   

Quinoline-based mol­ecules have shown significant promise in the development of clinically viable anti-cancer drugs (Afzal *et al.*, 2015 ▸). Metal complexes containing quinoline include: (i) square-planar palladium- and platinum-quinoline compounds, such as *trans*-[Pd(II)Cl_2_(quinoline)_2_], *cis*-[Pt(II)Cl_2_(quinoline)_2_] and *trans*-[Pd(II)(N_3_)_2_(quinoline)_2_] (Ha, 2012 ▸; Klapötke *et al.*, 2000 ▸; Raven *et al.*, 2012 ▸; Davies *et al.*, 2001 ▸), as well as (ii) tetra­hedral cobalt-, nickel- and zinc-quinoline compounds, of the form [*M* ^II^Cl_2_(quinoline)_2_] (Golic & Mirceva, 1988 ▸). Inter­estingly, despite the fact that the inter­action of dimethyl sulfoxide (Me_2_SO) with metal ions has been studied for many years (Cotton & Francis, 1960 ▸), metal compounds that incorporate both coordinated quinoline and Me_2_SO are rare, as illustrated by the fact that only one structurally characterized example is listed in the Cambridge Structural Database (Groom *et al.*, 2016 ▸), Zn(O_2_CC_6_H_4_C_2_HN_3_CO_2_CH_3_)_2_·(quinoline)·Me_2_SO (Ma *et al.*, 2012 ▸). Herein, we describe the structure of the complex salt [Co^II^(Me_2_SO)_6_][Co^II^Cl_3_quinoline]_2_, which can be obtained by the reaction of anhydrous cobalt(II) chloride with quinoline in Me_2_SO.
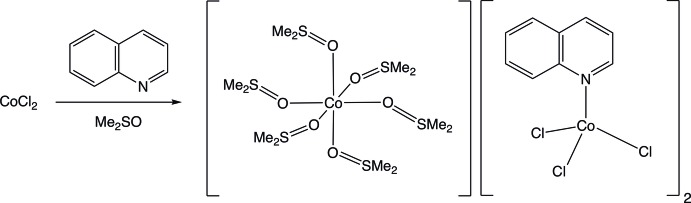


## Structural commentary   

**Figure 1:**
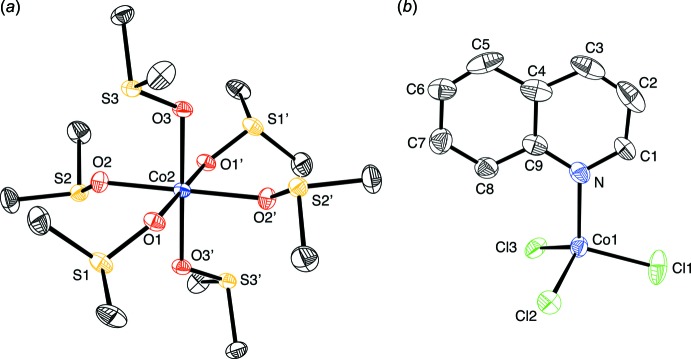
The mol­ecular structure of the complex salt [Co^II^(Me_2_SO)_6_][Co^II^Cl_3_quinoline]_2_, showing (*a*) the [Co^II^(Me_2_SO)_6_]^2+^ cation (primed labels are related by the symmetry code: −*x*, −*y*, −*z* + 2), and (*b*) the symmetry-unique [Co^II^Cl_3_quinoline]^−^ anion.

The mol­ecular structures of the cation and anion portions of the title complex are shown in Fig. 1 ▸ *a* and 1*b*, respectively. In the cation portion of this compound, the cobalt atom lies on a crystallographic inversion center and is coordinated to oxygen atoms of six Me_2_SO groups in an octa­hedral configuration. The cation is not rigorously octa­hedral, as the Co—O bond distances are slightly elongated in the axial positions [2.1258 (17) Å] compared to the equatorial positions [2.0606 (17)–2.0819 (18) Å], giving an average Co—O distance of 2.089 Å. A closely related complex, [Co(Me_2_SO)_6_][CoCl_4_], contains a cobalt cation that is similarly surrounded by six oxygen atoms in a slightly distorted octa­hedral configuration with Co—O distances between 2.06 (1) and 2.10 (1) Å, with a mean Co—O distance of 2.08Å (Ciccarese *et al.*, 1993 ▸). The O—Co—O (*cis*) bond angles in the title complex are close to 90°, ranging from 86.29 (7) to 93.71 (7)°, compared to 87.9 (5) to 90.8 (4)° in [Co(Me_2_SO)_6_][CoCl_4_] (Ciccarese *et al.*, 1993 ▸).

The cobalt atom in the anion portion of the title complex is attached to three chloro ligands and one quinoline moiety in a tetra­hedral arrangement. The Co—Cl bond distances range from 2.2517 (10) to 2.2534 (10) Å, with an average Co—Cl distance of 2.252 Å, while the Co—N distance is 2.054 (3) Å. The Cl—Co—Cl angles range from 108.21 (5) to 114.26 (4)°, giving an average of 110.98°, and the average N—Co—Cl angle is 107.88° [range 107.09 (9) to 108.80 (8)°], indicating that while the anion is close to tetra­hedral, there is some distortion. Inter­estingly, the [CoCl_4_]^2−^ anion in [Co(Me_2_SO)_6_][CoCl_4_] also showed some distortion with Co—Cl distances ranging from 2.265 (6) to 2.305 (7) Å, giving an average Co—Cl distance of 2.284 (6) Å, and the Cl—Co—Cl angles ranging from 107.1 (2) to 112.4 (2)° (Ciccarese *et al.*, 1993 ▸). The deviations from 109.5° in [Co(Me_2_SO)_6_][CoCl_4_] were ascribed to disorder, as indicated by the high anisotropic motion (Ciccarese *et al.*, 1993 ▸).

The degree of distortion from a tetra­hedral arrangement can be readily qu­anti­fied by the τ_4_ index that is reported and discussed elsewhere (Yang *et al.*, 2007 ▸, Palmer *et al.*, 2015 ▸). Briefly, τ_4_ is obtained from the expression, τ_4_ = [360 − (α + β)]/141, where α and β represent the two largest angles; a τ_4_ value of 1.00 indicates an idealized tetra­hedral geometry, whereas a value of 0.00 indicates an idealized square-planar geometry. In the title complex, α = 114.26 (4)° and β = 110.46 (4)°, such that τ_4_ is 0.96, which indicates very little deviation from a tetra­hedral geometry. For comparison, τ_4_ for the [CoCl_4_]^2−^ anion in [Co(Me_2_SO)_6_][CoCl_4_] is 0.98 (where α = 112.38° and β = 108.81°; Ciccarese *et al.*, 1993 ▸).

## Supra­molecular features   

**Figure 2:**
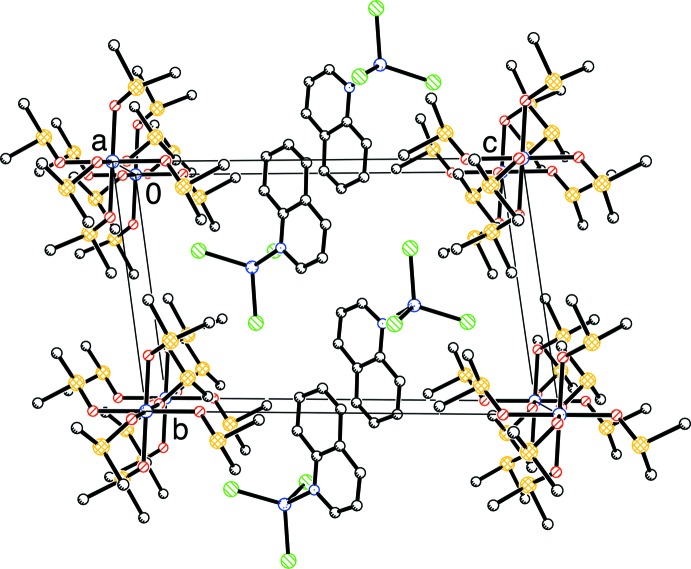
The packing of [Co^II^(Me_2_SO)_6_][Co^II^Cl_3_quinoline]_2_. H atoms have been omitted for clarity.

Fig. 2 ▸ shows the packing in the unit cell. There are no significant inter­molecular inter­actions between the [Co^II^(Me_2_SO)_6_]^2+^ and [Co^II^Cl_3_quinoline]^−^ ions, with the exception of very weak C—H⋯Cl interactions. The distances between the Cl and the carbon atoms of the methyl groups of the Me_2_SO ligands are, for example, Cl1⋯C32—S3 (symmetry code: *x*, *y*, *z*) [3.525 (3) Å], Cl1⋯C31—S3 (symmetry code: *x*, *y*, *z*) [3.736 (4) Å], Cl2⋯C22—S2 (symmetry code: 1 + *x*, 1 + *y*, *z*) [3.633 (4) Å], Cl2⋯C21—S2 (symmetry code: 1 + *x*, 1 + *y*, *z*) [3.770 (4) Å], Cl3⋯C12—S1 (symmetry code: 1 + *x*, *y*, *z*) [3.638 (4) Å] and Cl3⋯C32—S3 (symmetry code: *x*, 1 + *y*, *z*) [3.819 (4) Å] and are comparable to the sum of the van der Waals radii of Cl and CH_3_ of 3.80 Å (Pauling, 1986 ▸).

## Database survey   

The structure reported herein is closely related to the previously reported [Co(Me_2_SO)_6_][CoCl_4_] complex as discussed above (Ciccarese *et al.*, 1993 ▸). Inter­estingly, as long ago as 1960, and based on spectral and magnetic evidence only, Cotton & Francis reported that a complex having the empirical formula CoCl_2_·3Me_2_SO is more correctly formulated as [Co(Me_2_SO)_6_][CoCl_4_] (Cotton & Francis, 1960 ▸).

In addition to [Co(Me_2_SO)_6_][CoCl_4_], there are a few other examples of cobalt complexes solvated by Me_2_SO that are listed in the Cambridge Database (CSD Version 5.38; Groom *et al.*, 2016 ▸). For example, there are two reports for [Co(Me_2_SO)_6_][ClO_4_]_2_, one of which possesses Co—O distances in the range 2.0833 (17)–2.0934 (15) Å, giving a mean Co—O distance of 2.088 (5) Å, with O—Co—O (*cis*) angles between 90.11 (6) and 92.31 (6)° (Comuzzi *et al.*, 2002 ▸), while a subsequent report lists Co—O distances in the range 2.088 (2)–2.110 (2) Å, with O—Co—O (*cis*) angles between 85.26 (7) and 93.67 (8)° (Chan *et al.*, 2004 ▸). In [Co(Me_2_SO)_6_][SnCl_6_], both the cobalt and tin metal ions display an octa­hedral environments, with the Co—O bond lengths reported between 2.093 (4) and 2.113 (5) Å (White *et al.*, 2007 ▸). The O—Co—O (*cis*) angles vary between 89.0 (2) and 90.0 (2)° (White *et al.*, 2007 ▸).

In addition to the above Co^II^ compounds, the octa­hedral Co^III^ complex [Co(Me_2_SO)_6_][NO_3_]_3_ is also known and possesses six equivalent Co—O bond lengths of 2.005 (2) Å, which are shorter than the values in the Co^II^ complexes (Li & Ng, 2010 ▸).

Although Me_2_SO is typically coordinated to a metal *via* the oxygen atom (Sipos *et al.*, 2015 ▸; Calligaris, 2004 ▸; Calligaris & Carugo, 1996 ▸), there are examples in which Me_2_SO serves as an S-donor, as illustrated by the ruthenium complex [*mer*-RuCl_3_(acv)(Me_2_SO-S)(C_2_H_5_OH)]·C_2_H_5_OH (acv = a­cyclo­vir) (Turel *et al.*, 2004 ▸). With regard to cobalt, it has been noted that Co^II^ is a hard acceptor preferring hard-donor atoms like oxygen in Me_2_SO, the bonds being mainly electrostatic in nature (Comuzzi *et al.*, 2002 ▸). Nevertheless, while Me_2_SO coordination to cobalt through the soft-donor sulfur atom (rather than the oxygen atom) is rare, there are some notable examples. For example, the compound bis­(dimethyl sulfoxide)­hydridobis(tri­phenyl­phosphane)cobalt(I), [CoH(C_18_H_15_P)_2_(Me_2_SO)_2_], contains Co^I^ coordinating a hydride anion, two phosphine ligands and two Me_2_SO moieties that are bound through the sulfur atom in a distorted trigonal–bipyramidal structure (Hapke *et al.*, 2010 ▸). Inter­estingly, there is an example of a cobalt(III) porphyrin complex that contains both oxygen- and sulfur-bound Me_2_SO moieties, *i.e.* bis­(dimethyl sulfoxide-κ*O*)-(5,10,15,20-tetra­kis­(4-meth­oxy­phen­yl)porphyrinato)-cobalt(III) bis­(dimethyl sulfoxide-κ*S*)-(5,10,15,20-tetra­kis­(4-meth­oxy­phen­yl)porph­yr­inato)cobalt(III) bis­(hexa­fluoro­anti­monate) dimethyl sulfoxide solvate (Venkatasubbaiah *et al.*, 2011 ▸). The existence of both forms of Me_2_SO bonding to Co^III^ in this latter complex cannot be predicted readily by the application of traditional hard/soft-acid/base theory.

The Co—N bond length in the anion [Co^II^Cl_3_(quinoline)_2_]^−^ of the title compound is 2.037 (5) Å while the Co–Cl bond lengths are 2.2517 (10)–2.2534 (10) Å, and the Cl—Co—Cl and Cl—Co—N angles range between 108.21 (5) and 114.26 (4)°, and 107.09 (9) and 108.80 (8)°, respectively. For comparison, the Co—N bond lengths in the Co^II^Cl_2_(quinoline)_2_ complex are 2.061 (3) and 2.037 (5) Å and the Co—Cl bond lengths are 2.246 (2) and 2.241 (1) Å (Golic & Mirceva, 1988 ▸), while the Cl—Co—Cl angle is 114.5 (1)° and the Cl—Co—N angles range between 106.2 (1) and 108.9 (1)°.

## Synthesis and crystallization   

Anhydrous cobalt(II) chloride (97%; 0.1301 g, 0.0010 mol) was mixed with quinoline, C_9_H_7_N, (99%; 0.2595 g, 0.0020 mol) in Me_2_SO (20 mL) and refluxed for one h. After cooling down, the mixture was transferred to a beaker and placed in a desiccator containing anhydrous calcium chloride pellets (4–20 mesh) to crystallize over a period of four months. Deep-blue crystals of [Co(Me_2_SO)_6_]^2+^{[CoCl_3_quinoline]_2_}^−^ suitable for X-ray diffraction were obtained from this process of slow evaporation. Notably, when the reaction between anhydrous cobalt(II) chloride and quinoline is conducted in EtOH, rather than Me_2_SO, the previously reported [Co^II^Cl_2_(quinoline)_2_] complex is obtained (Golic & Mirceva, 1988 ▸).

## Refinement   

Crystal data, data collection and structure refinement details are summarized in Table 1 ▸. Hydrogen atoms on carbon were placed in calculated positions (C—H = 0.95–1.00 Å) and included as riding contributions with isotropic displacement parameters *U*_iso_(H) = 1.2*U*_eq_(C*sp* ^2^) or 1.5*U*_eq_(C*sp* ^3^).

## Supplementary Material

Crystal structure: contains datablock(s) I. DOI: 10.1107/S2056989018001652/lh5868sup1.cif

Structure factors: contains datablock(s) I. DOI: 10.1107/S2056989018001652/lh5868Isup2.hkl

CCDC reference: 1820336

Additional supporting information:  crystallographic information; 3D view; checkCIF report

## Figures and Tables

**Table 1:** Experimental details

Crystal data	
Chemical formula	[Co(C_2_H_6_OS)_6_][CoCl_3_(C_9_H_7_N)]_2_
*M*_r_	1116.57
Crystal system, space group	Triclinic, *P* 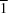
Temperature (K)	230
*a*, *b*, *c* (Å)	8.3182 (13), 9.6130 (15), 15.595 (2)
α, β, γ (°)	81.767 (2), 82.776 (2), 87.183 (2)
*V* (Å^3^)	1223.7 (3)
*Z*	1
Radiation type	Mo *K*α
μ (mm^−1^)	1.63
Crystal size (mm)	0.39 × 0.12 × 0.05

Data collection	
Diffractometer	Bruker APEXII CCD
Absorption correction	Multi-scan (*SADABS*; Bruker, 2008 ▸)
*T*_min_, *T*_max_	0.626, 0.746
No. of measured, independent and observed [*I* > 2σ(*I*)] reflections	19375, 7447, 4839
*R*_int_	0.035
(sin θ/λ)_max_ (Å^−1^)	0.715

Refinement	
*R*[*F* ^2^ > 2σ(*F* ^2^)], *wR*(*F* ^2^), *S*	0.045, 0.116, 1.02
No. of reflections	7447
No. of parameters	247
H-atom treatment	H-atom parameters constrained
Δρ_max_, Δρ_min_ (e Å^−3^)	0.77, −0.47

Computer programs: *APEX2* and *SAINT* (Bruker, 2008 ▸), *SHELXS97* and *SHELXTL* (Sheldrick 2008 ▸) and *SHELXL2014* (Sheldrick, 2015 ▸).

## supplementary crystallographic information

### Crystal data

**Table:**

[Co(C_2_H_6_OS)_6_][CoCl_3_(C_9_H_7_N)]_2_	*Z* = 1
*M**_r_* = 1116.57	*F*(000) = 571
Triclinic, *P*1	*D*_x_ = 1.515 Mg m^−^^3^
*a* = 8.3182 (13) Å	Mo *K*α radiation, λ = 0.71073 Å
*b* = 9.6130 (15) Å	Cell parameters from 7471 reflections
*c* = 15.595 (2) Å	θ = 2.4–30.1°
α = 81.767 (2)°	µ = 1.63 mm^−^^1^
β = 82.776 (2)°	*T* = 230 K
γ = 87.183 (2)°	Block, blue
*V* = 1223.7 (3) Å^3^	0.39 × 0.12 × 0.05 mm

### Data collection

**Table:**

Bruker APEXII CCD diffractometer	4839 reflections with *I* > 2σ(*I*)
φ and ω scans	*R*_int_ = 0.035
Absorption correction: multi-scan (SADABS; Bruker, 2008)	θ_max_ = 30.5°, θ_min_ = 1.3°
*T*_min_ = 0.626, *T*_max_ = 0.746	*h* = −11→11
19375 measured reflections	*k* = −13→13
7447 independent reflections	*l* = −22→22

### Refinement

**Table:**

Refinement on *F*^2^	0 restraints
Least-squares matrix: full	Hydrogen site location: inferred from neighbouring sites
*R*[*F*^2^ > 2σ(*F*^2^)] = 0.045	H-atom parameters constrained
*wR*(*F*^2^) = 0.116	*w* = 1/[σ^2^(*F*_o_^2^) + (0.0457*P*)^2^ + 0.7087*P*] where *P* = (*F*_o_^2^ + 2*F*_c_^2^)/3
*S* = 1.02	(Δ/σ)_max_ < 0.001
7447 reflections	Δρ_max_ = 0.77 e Å^−^^3^
247 parameters	Δρ_min_ = −0.46 e Å^−^^3^

### Special details

**Table:**

Geometry. All esds (except the esd in the dihedral angle between two l.s. planes) are estimated using the full covariance matrix. The cell esds are taken into account individually in the estimation of esds in distances, angles and torsion angles; correlations between esds in cell parameters are only used when they are defined by crystal symmetry. An approximate (isotropic) treatment of cell esds is used for estimating esds involving l.s. planes.

### Fractional atomic coordinates and isotropic or equivalent isotropic displacement parameters (Å^2^)

**Table:**

	*x*	*y*	*z*	*U*_iso_*/*U*_eq_	
Co1	0.56334 (6)	0.57989 (4)	0.70199 (3)	0.04816 (13)	
Co2	0.0000	0.0000	1.0000	0.02575 (11)	
Cl1	0.56412 (18)	0.34385 (10)	0.71205 (7)	0.0874 (4)	
Cl2	0.81055 (11)	0.65246 (10)	0.64138 (7)	0.0647 (2)	
Cl3	0.48261 (13)	0.64884 (10)	0.83322 (6)	0.0659 (3)	
N	0.3997 (3)	0.6584 (3)	0.61758 (17)	0.0502 (6)	
S1	−0.13554 (8)	0.28530 (7)	0.90892 (5)	0.04024 (17)	
S2	−0.09070 (8)	−0.10708 (7)	0.82809 (5)	0.03584 (16)	
S3	0.31946 (7)	0.11911 (7)	0.89997 (4)	0.03205 (15)	
O1	−0.0198 (2)	0.22294 (17)	0.97373 (12)	0.0334 (4)	
O2	−0.0073 (2)	0.00114 (19)	0.86695 (12)	0.0366 (4)	
O3	0.2483 (2)	0.01632 (18)	0.97778 (12)	0.0340 (4)	
C1	0.3345 (5)	0.5732 (4)	0.5722 (3)	0.0657 (10)	
H1A	0.3652	0.4773	0.5809	0.079*	
C2	0.2274 (6)	0.6144 (7)	0.5145 (3)	0.0948 (17)	
H2A	0.1791	0.5472	0.4885	0.114*	
C3	0.1909 (5)	0.7490 (7)	0.4948 (3)	0.0865 (15)	
H3A	0.1210	0.7776	0.4522	0.104*	
C4	0.2547 (4)	0.8511 (5)	0.5364 (2)	0.0667 (11)	
C5	0.2245 (6)	1.0016 (6)	0.5210 (3)	0.0875 (15)	
H5A	0.1511	1.0396	0.4821	0.105*	
C6	0.2971 (4)	1.0822 (5)	0.5601 (2)	0.0621 (10)	
H6A	0.2807	1.1796	0.5447	0.074*	
C7	0.3967 (5)	1.0394 (4)	0.6224 (3)	0.0706 (11)	
H7A	0.4403	1.1045	0.6519	0.085*	
C8	0.4309 (4)	0.8990 (4)	0.6405 (2)	0.0610 (9)	
H8A	0.5021	0.8674	0.6820	0.073*	
C9	0.3626 (4)	0.8004 (4)	0.5987 (2)	0.0534 (8)	
C11	−0.0084 (5)	0.3546 (4)	0.8131 (2)	0.0638 (10)	
H11A	−0.0742	0.4082	0.7720	0.096*	
H11B	0.0482	0.2779	0.7866	0.096*	
H11C	0.0699	0.4154	0.8286	0.096*	
C12	−0.2066 (4)	0.4461 (3)	0.9459 (3)	0.0588 (9)	
H12A	−0.2738	0.4975	0.9047	0.088*	
H12B	−0.1149	0.5020	0.9504	0.088*	
H12C	−0.2699	0.4266	1.0026	0.088*	
C21	0.0638 (5)	−0.2325 (4)	0.8012 (2)	0.0618 (9)	
H21A	0.0252	−0.2929	0.7638	0.093*	
H21B	0.0920	−0.2890	0.8542	0.093*	
H21C	0.1587	−0.1844	0.7708	0.093*	
C22	−0.1126 (5)	−0.0229 (4)	0.7210 (2)	0.0601 (9)	
H22A	−0.1599	−0.0871	0.6892	0.090*	
H22B	−0.0071	0.0041	0.6910	0.090*	
H22C	−0.1828	0.0602	0.7242	0.090*	
C31	0.3992 (4)	0.2550 (3)	0.9467 (2)	0.0536 (8)	
H31A	0.4504	0.3230	0.9006	0.080*	
H31B	0.4788	0.2149	0.9844	0.080*	
H31C	0.3121	0.3012	0.9805	0.080*	
C32	0.5044 (3)	0.0349 (3)	0.8624 (2)	0.0442 (7)	
H32A	0.5610	0.0958	0.8142	0.066*	
H32B	0.4817	−0.0527	0.8429	0.066*	
H32C	0.5715	0.0156	0.9095	0.066*	

### Atomic displacement parameters (Å^2^)

**Table:**

	*U*^11^	*U*^22^	*U*^33^	*U*^12^	*U*^13^	*U*^23^
Co1	0.0638 (3)	0.0365 (2)	0.0456 (3)	−0.00097 (19)	−0.0139 (2)	−0.00423 (18)
Co2	0.0226 (2)	0.0228 (2)	0.0314 (3)	−0.00025 (17)	−0.00292 (18)	−0.00259 (18)
Cl1	0.1588 (12)	0.0370 (5)	0.0626 (6)	−0.0162 (6)	−0.0005 (6)	−0.0004 (4)
Cl2	0.0591 (5)	0.0601 (5)	0.0769 (6)	−0.0037 (4)	−0.0140 (4)	−0.0104 (4)
Cl3	0.0886 (7)	0.0612 (5)	0.0506 (5)	0.0280 (5)	−0.0216 (4)	−0.0151 (4)
N	0.0579 (16)	0.0548 (17)	0.0405 (14)	−0.0099 (13)	−0.0111 (12)	−0.0073 (12)
S1	0.0376 (4)	0.0294 (3)	0.0545 (4)	0.0028 (3)	−0.0158 (3)	−0.0006 (3)
S2	0.0322 (3)	0.0401 (4)	0.0366 (4)	−0.0029 (3)	−0.0049 (3)	−0.0090 (3)
S3	0.0250 (3)	0.0296 (3)	0.0396 (4)	−0.0002 (2)	−0.0032 (3)	0.0012 (3)
O1	0.0318 (9)	0.0231 (8)	0.0447 (11)	0.0014 (7)	−0.0088 (8)	0.0001 (7)
O2	0.0448 (11)	0.0338 (10)	0.0317 (10)	−0.0067 (8)	−0.0041 (8)	−0.0049 (8)
O3	0.0232 (8)	0.0339 (10)	0.0420 (10)	−0.0012 (7)	−0.0027 (7)	0.0039 (8)
C1	0.061 (2)	0.066 (2)	0.079 (3)	−0.0140 (18)	−0.024 (2)	−0.022 (2)
C2	0.082 (3)	0.143 (5)	0.074 (3)	−0.030 (3)	−0.008 (2)	−0.056 (3)
C3	0.069 (3)	0.147 (5)	0.050 (2)	−0.008 (3)	−0.023 (2)	−0.023 (3)
C4	0.051 (2)	0.112 (3)	0.0344 (18)	−0.003 (2)	−0.0007 (15)	−0.0031 (19)
C5	0.078 (3)	0.110 (4)	0.059 (3)	0.031 (3)	−0.009 (2)	0.030 (3)
C6	0.056 (2)	0.083 (3)	0.0409 (19)	−0.0063 (19)	−0.0049 (16)	0.0146 (18)
C7	0.074 (3)	0.057 (2)	0.077 (3)	−0.0079 (19)	−0.005 (2)	0.000 (2)
C8	0.062 (2)	0.061 (2)	0.059 (2)	−0.0091 (17)	−0.0185 (17)	0.0077 (17)
C9	0.0495 (18)	0.073 (2)	0.0360 (17)	−0.0071 (16)	−0.0040 (14)	−0.0008 (16)
C11	0.078 (2)	0.053 (2)	0.054 (2)	0.0094 (18)	−0.0079 (18)	0.0151 (16)
C12	0.0554 (19)	0.0396 (17)	0.083 (3)	0.0199 (15)	−0.0223 (18)	−0.0105 (17)
C21	0.074 (2)	0.0475 (19)	0.068 (2)	0.0225 (17)	−0.0182 (19)	−0.0216 (17)
C22	0.082 (3)	0.060 (2)	0.0423 (18)	0.0125 (18)	−0.0244 (17)	−0.0116 (16)
C31	0.065 (2)	0.0356 (16)	0.060 (2)	−0.0148 (15)	0.0069 (16)	−0.0121 (15)
C32	0.0398 (15)	0.0405 (16)	0.0468 (17)	0.0067 (12)	0.0089 (13)	−0.0023 (13)

### Geometric parameters (Å, º)

**Table:**

Co1—N	2.054 (3)	C4—C5	1.446 (6)
Co1—Cl1	2.2517 (10)	C5—C6	1.269 (6)
Co1—Cl2	2.2521 (11)	C5—H5A	0.9400
Co1—Cl3	2.2534 (10)	C6—C7	1.362 (5)
Co2—O3	2.0606 (17)	C6—H6A	0.9400
Co2—O3^i^	2.0607 (17)	C7—C8	1.362 (5)
Co2—O2^i^	2.0818 (18)	C7—H7A	0.9400
Co2—O2	2.0819 (18)	C8—C9	1.404 (5)
Co2—O1^i^	2.1258 (17)	C8—H8A	0.9400
Co2—O1	2.1258 (17)	C11—H11A	0.9700
N—C1	1.331 (4)	C11—H11B	0.9700
N—C9	1.383 (4)	C11—H11C	0.9700
S1—O1	1.5236 (18)	C12—H12A	0.9700
S1—C12	1.775 (3)	C12—H12B	0.9700
S1—C11	1.784 (4)	C12—H12C	0.9700
S2—O2	1.5127 (19)	C21—H21A	0.9700
S2—C21	1.772 (3)	C21—H21B	0.9700
S2—C22	1.776 (3)	C21—H21C	0.9700
S3—O3	1.5273 (19)	C22—H22A	0.9700
S3—C32	1.775 (3)	C22—H22B	0.9700
S3—C31	1.776 (3)	C22—H22C	0.9700
C1—C2	1.351 (6)	C31—H31A	0.9700
C1—H1A	0.9400	C31—H31B	0.9700
C2—C3	1.316 (7)	C31—H31C	0.9700
C2—H2A	0.9400	C32—H32A	0.9700
C3—C4	1.410 (6)	C32—H32B	0.9700
C3—H3A	0.9400	C32—H32C	0.9700
C4—C9	1.424 (5)		
			
N—Co1—Cl1	107.09 (9)	C4—C5—H5A	120.0
N—Co1—Cl2	107.76 (8)	C5—C6—C7	125.4 (4)
Cl1—Co1—Cl2	108.21 (5)	C5—C6—H6A	117.3
N—Co1—Cl3	108.80 (8)	C7—C6—H6A	117.3
Cl1—Co1—Cl3	110.46 (4)	C6—C7—C8	117.8 (4)
Cl2—Co1—Cl3	114.26 (4)	C6—C7—H7A	121.1
O3—Co2—O3^i^	180.0	C8—C7—H7A	121.1
O3—Co2—O2^i^	90.17 (7)	C7—C8—C9	121.6 (4)
O3^i^—Co2—O2^i^	89.83 (7)	C7—C8—H8A	119.2
O3—Co2—O2	89.83 (7)	C9—C8—H8A	119.2
O3^i^—Co2—O2	90.17 (7)	N—C9—C8	120.7 (3)
O2^i^—Co2—O2	180.00 (10)	N—C9—C4	121.2 (3)
O3—Co2—O1^i^	91.82 (7)	C8—C9—C4	118.0 (4)
O3^i^—Co2—O1^i^	88.19 (7)	S1—C11—H11A	109.5
O2^i^—Co2—O1^i^	86.29 (7)	S1—C11—H11B	109.5
O2—Co2—O1^i^	93.71 (7)	H11A—C11—H11B	109.5
O3—Co2—O1	88.18 (7)	S1—C11—H11C	109.5
O3^i^—Co2—O1	91.81 (7)	H11A—C11—H11C	109.5
O2^i^—Co2—O1	93.71 (7)	H11B—C11—H11C	109.5
O2—Co2—O1	86.29 (7)	S1—C12—H12A	109.5
O1^i^—Co2—O1	180.000 (19)	S1—C12—H12B	109.5
C1—N—C9	116.5 (3)	H12A—C12—H12B	109.5
C1—N—Co1	120.2 (3)	S1—C12—H12C	109.5
C9—N—Co1	123.0 (2)	H12A—C12—H12C	109.5
O1—S1—C12	103.99 (14)	H12B—C12—H12C	109.5
O1—S1—C11	105.22 (15)	S2—C21—H21A	109.5
C12—S1—C11	98.78 (18)	S2—C21—H21B	109.5
O2—S2—C21	105.12 (15)	H21A—C21—H21B	109.5
O2—S2—C22	103.28 (15)	S2—C21—H21C	109.5
C21—S2—C22	98.54 (18)	H21A—C21—H21C	109.5
O3—S3—C32	103.92 (12)	H21B—C21—H21C	109.5
O3—S3—C31	104.93 (13)	S2—C22—H22A	109.5
C32—S3—C31	98.99 (16)	S2—C22—H22B	109.5
S1—O1—Co2	117.12 (10)	H22A—C22—H22B	109.5
S2—O2—Co2	124.52 (11)	S2—C22—H22C	109.5
S3—O3—Co2	118.17 (10)	H22A—C22—H22C	109.5
N—C1—C2	124.9 (4)	H22B—C22—H22C	109.5
N—C1—H1A	117.6	S3—C31—H31A	109.5
C2—C1—H1A	117.6	S3—C31—H31B	109.5
C3—C2—C1	119.7 (4)	H31A—C31—H31B	109.5
C3—C2—H2A	120.2	S3—C31—H31C	109.5
C1—C2—H2A	120.2	H31A—C31—H31C	109.5
C2—C3—C4	121.2 (4)	H31B—C31—H31C	109.5
C2—C3—H3A	119.4	S3—C32—H32A	109.5
C4—C3—H3A	119.4	S3—C32—H32B	109.5
C3—C4—C9	116.3 (4)	H32A—C32—H32B	109.5
C3—C4—C5	126.7 (4)	S3—C32—H32C	109.5
C9—C4—C5	117.0 (4)	H32A—C32—H32C	109.5
C6—C5—C4	120.0 (4)	H32B—C32—H32C	109.5
C6—C5—H5A	120.0		
			
C12—S1—O1—Co2	148.84 (16)	C4—C5—C6—C7	−5.0 (7)
C11—S1—O1—Co2	−107.81 (16)	C5—C6—C7—C8	4.8 (6)
C21—S2—O2—Co2	−95.18 (18)	C6—C7—C8—C9	−1.9 (6)
C22—S2—O2—Co2	162.00 (16)	C1—N—C9—C8	−177.3 (3)
C32—S3—O3—Co2	−145.35 (14)	Co1—N—C9—C8	−3.1 (4)
C31—S3—O3—Co2	111.19 (15)	C1—N—C9—C4	2.5 (5)
C9—N—C1—C2	−4.9 (6)	Co1—N—C9—C4	176.7 (2)
Co1—N—C1—C2	−179.3 (4)	C7—C8—C9—N	179.6 (3)
N—C1—C2—C3	5.5 (7)	C7—C8—C9—C4	−0.3 (5)
C1—C2—C3—C4	−3.5 (8)	C3—C4—C9—N	−0.9 (5)
C2—C3—C4—C9	1.4 (6)	C5—C4—C9—N	−179.7 (3)
C2—C3—C4—C5	−180.0 (4)	C3—C4—C9—C8	178.9 (3)
C3—C4—C5—C6	−176.3 (4)	C5—C4—C9—C8	0.2 (5)
C9—C4—C5—C6	2.3 (6)		

Symmetry code: (i) −*x*, −*y*, −*z*+2.
